# Natural and Undetermined Sudden Death: Value of Post-Mortem Genetic Investigation

**DOI:** 10.1371/journal.pone.0167358

**Published:** 2016-12-08

**Authors:** Olallo Sanchez, Oscar Campuzano, Anna Fernández-Falgueras, Georgia Sarquella-Brugada, Sergi Cesar, Irene Mademont, Jesus Mates, Alexandra Pérez-Serra, Monica Coll, Ferran Pico, Anna Iglesias, Coloma Tirón, Catarina Allegue, Esther Carro, María Ángeles Gallego, Carles Ferrer-Costa, Anna Hospital, Narcís Bardalet, Juan Carlos Borondo, Albert Vingut, Elena Arbelo, Josep Brugada, Josep Castellà, Jordi Medallo, Ramon Brugada

**Affiliations:** 1 Cardiovascular Genetics Center, University of Girona-IDIBGI, Girona (Spain); 2 Department of Medical Sciences, School of Medicine, University of Girona, Girona (Spain); 3 Cardiovascular Genetics Unit, Hospital Josep Trueta, Girona (Spain); 4 Arrhythmia Unit, Hospital Sant Joan de Déu, University of Barcelona, Barcelona (Spain); 5 Forensic Pathology Service, Institut Medicina Legal Ciències Mèdiques Catalunya, Barcelona (Spain); 6 Gendiag SL, Barcelona (Spain); 7 Forensic Pathology Service, Institut Medicina Legal i Ciències Forenses de Catalunya, Girona (Spain); 8 Histopathology Unit, Instituto Nacional de Toxicología y Ciencias Forenses, Barcelona (Spain); 9 Arrhythmia Unit, Hospital Clinic de Barcelona, University of Barcelona, Barcelona (Spain); Pennsylvania State University, UNITED STATES

## Abstract

**Background:**

Sudden unexplained death may be the first manifestation of an unknown inherited cardiac disease. Current genetic technologies may enable the unraveling of an etiology and the identification of relatives at risk. The aim of our study was to define the etiology of natural deaths, younger than 50 years of age, and to investigate whether genetic defects associated with cardiac diseases could provide a potential etiology for the unexplained cases.

**Methods and Findings:**

Our cohort included a total of 789 consecutive cases (77.19% males) <50 years old (average 38.6±12.2 years old) who died suddenly from non-violent causes. A comprehensive autopsy was performed according to current forensic guidelines. During autopsy a cause of death was identified in most cases (81.1%), mainly due to cardiac alterations (56.87%). In unexplained cases, genetic analysis of the main genes associated with sudden cardiac death was performed using Next Generation Sequencing technology. Genetic analysis was performed in suspected inherited diseases (cardiomyopathy) and in unexplained death, with identification of potentially pathogenic variants in nearly 50% and 40% of samples, respectively.

**Conclusions:**

Cardiac disease is the most important cause of sudden death, especially after the age of 40. Close to 10% of cases may remain unexplained after a complete autopsy investigation. Molecular autopsy may provide an explanation for a significant part of these unexplained cases. Identification of genetic variations enables genetic counseling and undertaking of preventive measures in relatives at risk.

## Introduction

Natural death defines the death primarily attributed to an illness or an internal malfunction of the body, and not directly influenced by external forces. The forensic pathologists can straightforwardly identify the cause of natural death when macroscopic investigations are conclusive [1]. However, when a macroscopic cause is not evident, the final identification of causality can become tedious and complicated. Despite comprehensive macroscopic, microscopic as well as toxicological investigation, around 5%-10% of cases will remain unexplained and will be classified as sudden unexpected deaths (SUD), often defined in the report as *death from a supposed arrhythmia* [2, 3]. In the young population, this percentage may increase up to 30%-50% [4–6]. Even if the cause of death remains unanswered after a thorough forensic investigation, the legal work is usually concluded. However, from the medical standpoint, an unidentified etiology conveys dangerous clinical implications; these unexplained deaths may be caused by an inherited cardiac disease, which potentially leaves family members at risk.

In a simplistic classification, deaths caused by cardiac genetic alterations may affect two different disease groups, channelopathies and cardiomyopathies [7]. It is estimated that 10% to 25% of SUD in the adult, and up to one-third in infantile and juvenile SUD, may be explained by cardiac channelopathies [8–11]. These channelopathies include mainly Long QT syndrome (LQTS), Short QT syndrome (SQTS), Catecholaminergic Polymorphic Ventricular Tachycardia (CPVT), and Brugada syndrome (BrS) [12]. In addition, pathogenic variations in genes encoding structural proteins are responsible for cardiomyopathies (Hypertrophic Cardiomyopathy (HCM), Dilated Cardiomyopathy (DCM), and Arrhythmogenic Cardiomyopathy (AC), among others). These cardiomyopathies will usually present anatomo-morphological changes in the cardiac tissue, which can be diagnosed at autopsy [13], but recent reports have suggested that in infants they could also be potentially responsible for sudden death in the structurally normal heart [14, 15].

Because SUD may be the first manifestation of an unknown inherited cardiac disease, the use of genetic testing, the so-called molecular autopsy, could be determinant in the discovery of causality, in the identification of genetic carriers in family members, and in the further adoption of preventive strategies [16, 17]. Despite that current forensic guidelines recommend molecular autopsy as part of routine protocol in SUD cases, this is seldom performed[18, 19]. This molecular investigation has been mainly limited to research projects, and usually constrained to the analysis of the most prevalent genes associated with channelopathies *(KCNQ1*, *KCNH2*, *SCN5A* and *RYR2)*, leaving several potential candidate genes untested [20, 21]. With the advent of high-throughput genetic technologies, Next Generation Sequencing (NGS), massive genetic sequencing has become available [22]. Recent reports have shown that NGS analysis could become an important asset in post-mortem examination [23–27]. To date, only one comprehensive study has been performed to prove the value of genetic testing in natural death [28].

In the present work we have addressed this issue by performing a prospective full epidemiological analysis of sudden death in a correlative cohort of SUD victims younger than 50 years of age, referred for forensic investigation due to out of hospital natural death. The goal was to define the etiology of natural death in the young, and to investigate whether genetic defects could contribute to this event. To perform the genetic analysis we have taken advantage of a custom-made resequencing panel. By including molecular diagnostic strategies, the ultimate goal of this work has been to develop a decision algorithm to better refine the forensic investigation, to assess the value of this powerful diagnostic tool in detecting a potential etiology, and to define which families would benefit from further clinical and genetic investigation.

## Methods

Our project was initiated in 2012, in collaboration with Institut de Medicina Legal i Ciències Forenses de Catalunya (IMLCFC). The IMLCFC oversees and concentrates all SUD cases, which require forensic investigation. We have focused the project in those cases investigated by the pathologists in the Catalonia area (population of 7.5 million people).

### Forensic analysis

The study was approved by the ethics committee of our Hospital, and follows the Helsinki II declaration. Our inclusion criteria were victims of sudden death, from natural cause, younger than 50 years of age. A complete autopsy examination was performed according to current international regulations [1, 18]. When the macroscopic autopsy was labelled as negative, the forensic pathologists performed complete histological and toxicological investigation, and collected a blood sample for genetic investigation. We excluded those cases in which the autopsy was labelled as violent death, including death from drug overdose.

### DNA sample

Genomic DNA was extracted with Chemagic MSM I from post-mortem whole blood (Chemagic human blood). DNA was checked in order to assure quality (Absorbance 260/280:260/230 should be a minimum 1.8: 2.2 respectively), and was quantified before processing with the NGS strategy. Spectrophotometric measurements were performed to assess quality ratios of absorbance; DNA concentration was determined by fluorometry (Qubit, Life Technologies). DNA integrity was assessed on a 0.8% agarose gel.

### NGS sample preparation

The DNA was fragmented (Bioruptor, Diagenode). Library preparation was performed according to the manufacturer’s instructions (SureSelect XT Custom 0.5–2.9Mb library, Agilent Technologies, Inc). After capture, indexed libraries were sequenced in six-sample pools per cartridge. Paired-end sequencing process was developed on MiSeq System (Illumina) using 2x150 bp reads length.

### Custom Resequencing panel

Those samples with a good DNA quality were investigated using a custom-made genetic panel, which included 55 genes associated with SCD (*ACTC1*, *ACTN2*, *ANK2*, *CACNA1C*, *CACNB2*, *CASQ2*, *CAV3*, *CRYAB*, *CSRP3*, *DES*, *DMD*, *DSC2*, *DSG2*, *DSP*, *EMD*, *FBN1*, *GLA*, *GPD1L*, *HCN4*, *JPH2*, *JUP*, *KCNE1*, *KCNE2*, *KCNH2*, *KCNJ2*, *KCNQ1*, *LAMP2*, *LDB3*, *LMNA*, *MYBPC3*, *MYH6*, *MYH7*, *MYL2*, *MYL3*, *MYOZ2*, *PDLIM3*, *PKP2*, *PLN*, *PRKAG2*, *RYR2*, *SCN4B*, *SCN5A*, *SGCA*, *SGCB*, *SGCD*, *TAZ*, *TCAP*, *TGFB3*, *TGFBR2*, *TNNC1*, *TNNI3*, *TNNT2*, *TPM1*, *TTN*, and *VCL*). The panel also included structural proteins, as some recent publications have suggested that variants in these genes may be associated with SCD, even in the structurally normal heart [11]. All gene isoforms described in Ensembl 75 (http://www.ensembl.org/) which have been linked at least with either a RefSeq code (http://www.ncbi.nlm.nih.gov/refseq/) or CCDS (https://www.ncbi.nlm.nih.gov/CCDS/) were included. Coordinates of sequence data were based on UCSC human genome version hg19 (NCBI GRCh37 built). Biotinylated cRNA probe solution was used as a capture probe (Agilent Technologies). Probes were designed using eArray (Agilent Technologies) and the design was optimized by Gendiag.exe S.L. The gene panel final size was 432,512kbp. This custom enrichment gene design is commercialized by Ferrer inCode as SudD inCode®.

### Sanger sequencing

Sanger sequencing was used to confirm non-common (Minor Allele Frequency–MAF- < 1%) genetic variants detected by NGS, as well as in the genetic analysis of those cases with poor DNA quality. In this situation, we limited the analysis to the guideline recommended genes (*SCN5A* -NM_198056-, *KCNQ1* -NM_000218-, *KCNH2* -NM_000238-, *KCNE1* -NM_000219-, *KCNE2* -NM_172201-, and *RyR2* -NM_001035-) [29, 30]. The exons and exon-intron boundaries of each gene were amplified (Verities PCR, Applied Biosystems, Austin, TX, USA), the PCR products were purified (Exosap-IT, Affymetrix, Inc. USB® Products, Cleveland, OH, USA) and they were directly sequenced in both directions (Big Dye Terminator v3.1 and 3130XL Genetic Analyzer, both from Applied Biosystems) with posterior SeqScape Software v2.5 (Life Technologies) analysis, comparing obtained results with the reference sequence from hg19. The identified variations were compared with DNA sequences from 300 healthy Spanish individuals (individuals not related to any patient and of the same ethnicity; 600 alleles), as control cases, and contrasted with Human Gene Mutation Database -HGMD- (http://www.hgmd.cf.ac.uk/ac/index.php), HapMap (http://hapmap.ncbi.nlm.nih.gov/), 1000 genomes project (http://www.1000genomes.org/), Exome Aggregation Consortium–ExAC- (http://exac.broadinstitute.org/), and Exome Variant Server–EVS–(http://evs.gs.washington.edu/EVS/). Sequence variants were described following the HGVS rules (http://www.hgvs.org/), and checked in Mutalyzer (https://mutalyzer.nl/).

### Bioinformatics

The secondary bioinformatic analysis of the data obtained included adaptor and low quality bases trimming on FASTQ files. Trimmed reads were mapped with GEM III. The output were sorted and uniquely and properly mapped read pairs were selected. Finally, the variant calling over the cleaned BAM were performed with SAMtools v.1.2 together with an ad hoc developed script. The final annotation steps provided information included in public databases. Variants were annotated with dbSNP human build 142 IDs (http://www.ncbi.nlm.nih.gov/SNP/); the 1000 Genomes browser Phase 3 (http://www.1000genomes.org/); the Exome Aggregation Consortium (ExAC) v.0.3 (http://exac.broadinstitute.org/); NHLBI Exome Sequencing Project (ESP) ESP6500SI-V2 (http://evs.gs.washington.edu/EVS/); Ensembl information and in-home database IDs, if available. The Human Gene Mutation Database (HGMD, http://www.hgmd.cf.ac.uk/ac/index.php) was also consulted to identify previously reported pathogenic mutations. In silico prediction of pathogenicity of novel genetic variations was assessed in CONDEL software (CONsensus DELeteriousness scores of missense SNVs) (http://bg.upf.edu/condel/), Mutation Taster (http://www.mutationtaster.org/), and PROVEAN (Protein Variation Effect Analyzer) (http://provean.jcvi.org/index.php). Alignment of DNA sequences for different species was also performed for these novel variations using UniProt database (http://www.uniprot.org/).

### Assessment of pathogenicity

The rare variants (MAF < 1%) were classified according recent ACMG guidelines [31], following the criteria:

Likely/probably benign variants (PBV):

- Variants already described in any of databases, with all in-silico models predicted neutrality.

Variants of uncertain/unknown significance (VUS):

- Novel variants and all in-silico models predicted neutrality or differed between predictions.
- Variants already described in any of databases, and in-silico models differed between predictions.

Likely/probably pathogenic variants (PPV):

- Likely pathogenic variants reported to be disease-causing but where the author has indicated that there may be some degree of doubt, or subsequent evidence has come to light in the literature.
- Radical variants (insertions, deletions or premature stop codons)
- Splice site variants between ± 5 nucleotides, and all in-silico models consulted predicted pathogenicity.
- Novel variants with all in-silico models predicting pathogenicity.

Disease causing mutations (DM):

- Variants already reported to be disease-causing.

## Results

This is a three-year prospective study that was started on February 2012. We have collected a total of 789 consecutive cases -609 males (77.19%) and 180 females (22.81%)-. The range of age is from 0 to 50 years of age (average 38.6±12.2 years old). The average age is 39.3±11.2 years in males and 36.2±14.9 years in females. In order to classify the cases, we have divided the cohort in groups of 10 years (0–10, 11–20, 21–30, 31–40, 41–50 years old).

### Prevalence of natural death according to age

**Fig 1:**
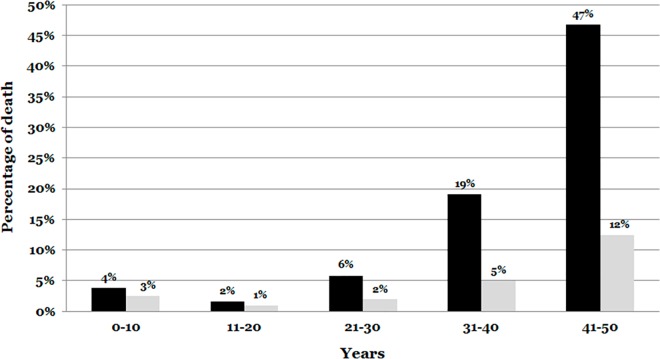
Percentage of natural death according to age and gender. The main percentage of death occurs in last range of ages. In all ranges of age, males are a high percentage of death. Males are indicated in black color. Females are indicated in white color.

In our cohort, most cases were between 41 and 50 years of age (467 out of 789, 59.19%). A total of 190 cases (24.08%) were between 31–40 years old and 132 cases (16.73%) were below age 30. Regarding gender differences, the number of males was higher in all ranges of age, showing most differences after age 30, with males nearing 80% of cases (Fig 1).

### Context in which the natural death took place

**Fig 2:**
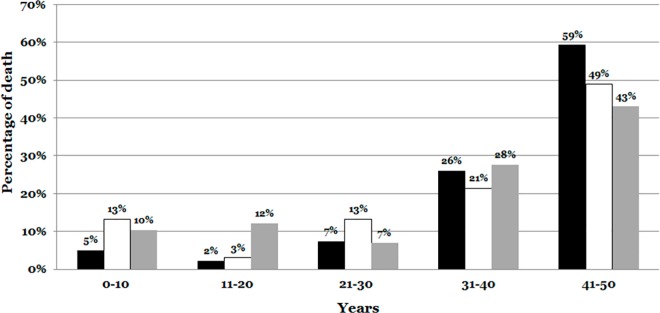
Context of death. The daily activities had a higher prevalence in cases higher 30 years old. Daily activities are indicated in black color. Sleep is indicated in white color. Exercise/Stress is indicated in grey color.

Deaths were classified depending whether they took place during stress/exercise, during sleep, or during routine daily activities. Information about the context of death was provided in 532 cases. The majority of deaths occurred during routine daily activities (376 cases, 70.68%), 98 occurred during sleep (18.42%) and 58 during exercise (10.90%). Deaths during sleep and during exercise were more common before the age of 20 (Fig 2).

### Etiology of death

Concerning the cause of death, the forensic pathologist directly determined a conclusive cause of death after macroscopic evaluation (positive macroscopic autopsy) in 506 cases (64.13%), while a yet inconclusive autopsy (negative macroscopic autopsy) was reported in the remaining cases. Most macroscopically positive cases were males (383 cases, 75.69%). Regarding negative autopsy cases, most of these cases were also males (226 cases, 79.86%).

### Positive macroscopic autopsy

Out of the 506 cases, the macroscopic investigation defined the following causes of death: cardiac in 230 cases (45.45%)–mainly coronary artery disease (127 cases)-; vascular (embolism or hemorrhage) in 137 cases (27.08%); pulmonary/respiratory in 91 cases (17.98%) -infectious process being responsible for the death in 47 cases, and digestive in 21 cases-; finally, 27 cases (5.34%), had other less common findings which included 8 non-vascular neurological, 7 carcinogenic, 6 endocrinologic, 2 obstetric, 1 infectious and 3 multiorganic failure.

### Negative macroscopic autopsy. Microscopic analyses

The macroscopic autopsy was not able to detect the cause of death in 283 cases (35.87%) and these were labeled as macroscopically negative. To identify a potential cause of death, these cases were further investigated with histological analysis. This investigation was able to refine the potential cause of death into the following subgroups: 1) Cardiac, which included coronary disease in 98 cases (34.63%) (presence of thrombus, of myocardial infarction or of severe coronary stenosis >75%), and 36 (12.72%) potentially cardiac inherited cases, by histological identification of cardiomyopathy; and, 2) unexplained cases, which included 149 (52.65%) cases with microscopic findings showing no histological alterations). Among these 149 cases, there were 23 cases (15.44%) in which death occurred before the first year of age, thus they were labeled as sudden infant death syndrome (SIDS).

### Natural death according to context and final autopsy results

**Fig 3:**
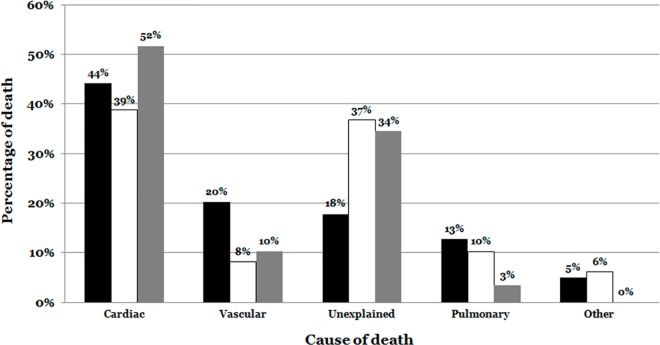
Cause of death according to context of death. The distribution showed that cardiac causes were more prevalent in the context of stress/exercise, while vascular were more prevalent during daily activities. The unexplained cases had a higher presence in context of sleep and stress/exercise. Daily activities are indicated in black color. Sleep is indicated in white color. Exercise/Stress is indicated in grey color.

With further inclusion of histological analysis, 364 out of 789 cases (46%) were definitively labeled as deaths from a cardiac origin. This percentage is underrepresented, as it did not include the 149 negative cases, some of whom presumably died also from cardiac causes. Stress/exercise related death was more frequent in cardiac cases (51.72%), while deaths during daily activity were more prevalent in vascular (20.23%), as well as pulmonary (12.84%) etiologies (Fig 3).

### Natural Death according to age groups

**Fig 4:**
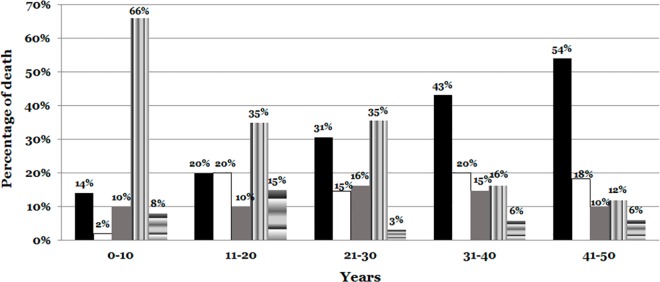
Cause of death distributed according to age. There is an increase of cardiac causes with age, reaching 54% of cases in the older group of age. However the unexplained cases were common in young below 30 years old, reaching the 66% of cases in youngest group of age. Cardiac is indicated in black color. Vascular is indicated in white color. Pulmonary is indicated in grey color. Unexplained is indicated in vertical lines of grey color. Other causes are indicated in horizontal lines of grey color.

We have divided the results in five groups of age (Fig 4):

#### 0–10 years old cohort

This sub-group included a total of 50 cases (6.34%). Of them, 7 cases (14%) died of cardiac causes (2 DCM, 2 myocarditis, 1 LQTS, 2 cardiac cause not specified), 1 case (2%) of neurological vascular causes, 5 cases (10%) of respiratory affectations (2 infectious, 1 aspiration, 1 asthmatic and 1 obstructive), 3 cases (6%) of cerebral/neurological (2 infectious and 1 malformation), and 1 case (2%) died still birth. Finally, 33 cases (66%) were left unexplained (23 SIDS and 1 SUDEP) after complete autopsy.

#### 11–20 years old cohort

This sub-group included 20 cases (2.53%). Of them, 4 (20%) died of cardiac causes (1 HCM, 1 DCM, 1 Congenital and 1 myocarditis), 4 (20%) of vascular causes (3 pulmonary and 1 digestive injuries), 2 (10%) of respiratory affectations (infectious), 1 (5%) of cerebral/neurological edema, and 2 (10%) of other causes (1 digestive and 1 endocrinology). Finally, 7 (35%) cases remained unexplained.

#### 21–30 years old cohort

This sub-group included 62 samples (7.86%). A total of 19 (30.65%) died of cardiac causes (6 coronary, 5 HCM, 1 DCM, 2 AC, 1 myocarditis, 1 congenital, 1 transplant, 1 valvular and 1 cardiac cause not specified), 9 (14.52%) of vascular injuries (5 pulmonary, 1 aortic, 2 neurological and 1 digestive injuries), 10 (16.13%) of respiratory affectations (4 infection, 3 edema, 2 aspiration and 1 asthmatic), 1 (1.61%) of cerebral/neurological pathologies (infection), and 1 (1.61%) of other causes (carcinogenic). Finally, 22 (35.48%) cases were unexplained.

#### 31–40 years old cohort

This included 190 samples (24.08%). Of them, 82 (43.16%) died of cardiac causes (49 coronary, 11 cardiac causes not specified, 6 DCM, 8 HCM, 1 AC, 3 myocarditis, 2 congenital and 2 fibrosis), 38 (20%) of vascular injuries (15 pulmonary, 9 neurological, 8 aortic and 6 digestive), 28 (14.74%) of respiratory affectations (12 infectious, 7 edema, 3 aspiration, 2 asthmatic and 4 obstructive), 3 (1.58%) of cerebral/neurological pathologies (2 infectious and 1 edema), and 8 (4.21%) of other causes (3 digestive, 4 carcinogenic and 1 multiorganic failure). Finally, 31 (16.32%) cases were unexplained.

#### 41–50 years old cohort

This included 467 samples (59.19%). Of them, 252 (53.96%) died of cardiac causes (171 coronary, 24 cardiac causes not specified, 18 DCM, 34 HCM, 2 AC, 2 Valvular, 1 pericarditis), 85 (18.20%) of vascular injuries (31 pulmonary, 28 digestive, 19 neurological, and 7 aortic), 46 (9.85%) of respiratory affectations (27 infectious, 10 edema, 6 obstructive, 1 asthmatic, 1 aspiration, and 1 hemorrhagic), and 28 (5.99%) of other causes (17 digestive, 7 carcinogenic, 1 endocrine, 1 multiorganic failure, 1 obstetric and 1 renal infectious causes). Finally, 56 (11.99%) cases were unexplained.

### Molecular autopsy

In addition to defining the etiology of natural death in our cohort, we wanted to assess whether the use of genetics could improve diagnosis ascertainment, and could better define which, if any, family members should undergo clinical/genetic evaluation. Thus, we focused our efforts in those forensic cases with potentially inherited disease or with negative microscopic autopsy:

### Potentially inherited subgroup

**Fig 5:**
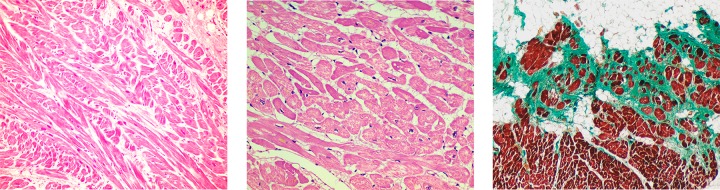
Representative microscopic images of samples showing cardiomyopathy. Left. Hypertrophic Cardiomyopathy (Hematoxylin-Eosin, 20x); Center. Dilated Cardiomyopathy (Hematoxylin-Eosin, 20x); Right. Arrhythmogenic Cardiomyopathy (Masson’s Trichrome, 10x).

This study was limited to cases previously considered macroscopically negative by the forensic pathologist but identified after histological analysis. A total of 32 samples showing histological alterations associated with cardiomyopathies (10 DCM, 19 HCM, 1 AC, and 2 fibrosis)(Fig 5) were screened by NGS method. The genetic screening identified a total of 62 rare variants in 25 out of 32 samples (78.13%). Twelve variants (19.35%) were novel. All cases carried at least one variant in genes codifying for structural proteins. However, 10 cases carried at least one additional rare variant in genes encoding proteins associated with ion channels or associated proteins. According to our classification criteria, 2 variants (3.23%) were considered PBV, 39 (62.90%) VUS, 12 (19.35%) PPV, and 9 (14.52%) DM (Table 1).

**Table 1:** Genetic data of variants identified, 26 positive samples from the 32 cardiomyopathies.

Range	ID	Proc.	Age	Gender	Autopsy	Gene	Nucleotide	Variant	dbSNP	MAF	ExAC %	HGMD	Classification	Disease	PPH2	Provean	Mut. Taster
11–20	1	NGS	17	M	HCM	*TNNI3*	c.611G>A	p.R204H	—	—	—	CM050764	DM	HCM	Damaging	Deleterious	Disease causing
11–20	2	NGS	19	M	DCM	*JUP*	c.56C>T	p.T19I	rs570878629	—	0.01139	CM098198	PPV	ARVC	Damaging	Deleterious	Disease causing
11–20	2					*VCL*	c.2046A>T	p.L682F	rs565398652	—	0.006589	—	VUS	—	Damaging	Neutral	Disease causing
11–20	2					*TTN*	c.81269T>C	p.I27090T	rs750026544	—	0.003331	—	PBV	—	Benign	Neutral	Polymorphism
11–20	2					*TTN*	c.26041A>G	p.I8681V	—	—	—	—	VUS	—	Damaging	Neutral	Disease causing
21–30	3	NGS	26	M	HCM	*SCN5A*	c.569G>A	p.R190Q	rs199473069	—	0.01131	CM077628	DM	LQTS	Damaging	Deleterious	Disease causing
21–30	4	NGS	26	M	HCM	*MYBPC3*	c.1505G>A	p.R502Q	rs397515907	—	—	CM981325	DM	HCM	Damaging	Deleterious	Disease causing
21–30	5	NGS	29	M	HCM	*TTN*	c.14818G>A	p.A4940T	rs72648947	0.0722/0/0.0493	0.04558	—	VUS	—	Damaging	Neutral	Tolerated
21–30	5					*TTN*	c.17441G>A	p.G5814D	rs72648964	0.1332/0.6309/0.2741	0.1708	—	VUS	—	Damaging	Neutral	Tolerated
21–30	5					*TTN*	c.47849A>G	p.K15950R	rs72646823	0.1332/0.359/0.2057	0.1372	—	VUS	—	Damaging	Neutral	Tolerated
21–30	6	NGS	30	M	HCM	*PKP2*	c.184C>A	p.Q62K	rs199601548	0.0358/0.0/0.0238	0.01679	CM061171	DM	ARVC	Damaging	Neutral	Disease causing
21–30	6					*MYBPC3*	c.1598A>G	p.Q533R	—	—	—	—	VUS	—	Benign	Neutral	Disease causing
21–30	6					*FBN1*	c.3058A>G	p.T1020A	rs111801777	0.0349/0.0/0.0231	0.04632	CM010035	PPV	Marfan	Benign	Neutral	Polymorphism
21–30	6					*TTN*	c.85109G>C	p.R28370T	—	—	—	—	PPV	—	Damaging	Deleterious	Disease causing
31–40	7	NGS	31	M	Fibrosis	*ANK2*	c.11326A>G	p.T3776A	rs746620876	—	—	—	VUS	—	—	Neutral	Polymorphism
31–40	7					*TTN*	c.90538C>T	p.R30180C	rs72648272	0.4066/0.0495/0.2902	0.4045	—	VUS	—	Damaging	Deleterious	Disease causing
31–40	7					*TTN*	c.26542C>T	p.H8848Y	rs72650011	0.4067/0.0745/0.2987	0.4143	CM116750	DM	ARVC	Benign	Deleterious	Polymorphism
31–40	7					*TTN*	c.9359G>A	p.R3120Q	rs72647894	0.4419/0.0908/0.3229	0.4396	—	VUS	—	Damaging	Neutral	Polymorphism
31–40	8	NGS	31	M	Fibrosis	*ANK2*	c.9185A>G	p.E3062G	—	—	—	—	PPV	—	Damaging	Deleterious	Disease causing
31–40	8					*TTN*	c.100184delA	p.K3395fs	rs281864930	—	—	—	PPV	—	—	—	Disease causing
31–40	9	NGS	37	M	HCM	*SCN5A*	c.1127G>A	p.R376H	rs199473101	0.0119/0/0.008	—	CM054856	PPV	BrS	Damaging	Neutral	Disease causing
31–40	9					*TNNT2*	c.101C>T	p.A34V	—	—	0.0008242	—	VUS	—	—	Neutral	Polymorphism
31–40	9					*TTN*	c.89795C>A	p.P29932H	—	—	—	—	PPV	—	Damaging	Deleterious	Disease causing
31–40	9					*TTN*	c.46610G>A	p.R15537H	—	—	0.001666	—	VUS	—	Damaging	Deleterious	Disease causing
31–40	10	NGS	38	M	DCM	*ACTN2*	c.1984C>T	p.R662W	rs150021739	0.0/0.0227/0.0077	0.004943	—	VUS	—	Damaging	Deleterious	Disease causing
31–40	11	NGS	38	M	HCM	*MYBPC3*	c.2539_2549delinG	p.Y847_N850delinD	—	—	—	—	PPV	—	—	—	—
31–40	11					*TTN*	c.34372G>C	p.V11458L	—	—	—	—	VUS	—	Damaging	Neutral	Disease causing
31–40	12	NGS	39	M	HCM	*DSG2*	c.3175T>A	p.S1059T	rs201786158	—	0.02816	CM071712	DM	ARVC	Damaging	Neutral	Polymorphism
31–40	12					*MYBPC3*	c.103C>T	p.R35W	—	—	0.005626	CM0910203	DM	HCM	Damaging	Deleterious	Disease causing
31–40	12					*TTN*	c.15625G>A	p.G5209S	rs374964612	0.0121/0.0/0.0083	0.005090	—	VUS	—	Damaging	Deleterious	Disease causing
31–40	13	NGS	40	M	HCM	*TTN*	c.76559G>A	p.S25520N	rs200450022	0.085/0.0/0.0583	0.05921	—	VUS	—	Bening	Neutral	Disease causing
31–40	13					*TTN*	c.17066G>C	p.G5689A	rs200118743	0.0843/0.0/0.0574	0.08920	—	VUS	—	Bening	Neutral	Disease causing
41–50	14	NGS	41	M	DCM	*MYBPC3*	c.2177C>T	p.R726C	—	—	0.003633	CM092563	DM	HCM	Damaging	Deleterious	Disease causing
41–50	14					*HCN4*	c.2800C>T	p.R934C	rs199638465	0.0133/0/0.0088	0.06047	—	VUS	—	Damaging	Neutral	Disease causing
41–50	15	NGS	41	M	DCM	*DSP*	c.6497G>A	p.R2166Q	—	—	0.0008244	—	VUS	—	Benign	Neutral	Polymorphism
41–50	15					*TTN*	c.47501T>G	p.I15834S	rs776899398	—	0.0008312	—	VUS	—	Damaging	Deleterious	Disease causing
41–50	15					*TTN*	c.4208G>C	p.R1403T	rs531590921	—	0.03321	—	VUS	—	Damaging	Deleterious	Disease causing
41–50	16	NGS	44	M	HCM	*CSRP3*	c.10T>C	p.W4R	rs45550635	0.5358/0.0455/0.3697	0.237	CM023060	DM	DCM	Damaging	Deleterious	Disease causing
41–50	16					*HCN4*	c.2730C>A	p.F910L	rs200814534	0.0123/0.0/0.0081	0.01675	—	VUS	—	Benign	Neutral	Disease causing
41–50	17	NGS	45	M	HCM	*ANK2*	c.10948G>C	p.E3650Q	—	—	0.001649	—	VUS	—	Benign	Neutral	Disease causing
41–50	17					*TTN*	c.19570G>A	p.D6524N	rs72648973	0.1463/0.0539/0.1175	0.07463	—	VUS	—	Damaging	Deleterious	Disease causing
41–50	17					*TTN*	c.41023C>T	p.P13675S	rs72677242	0.3262/0.0258/0.2305	0.5239	—	VUS	—	Benign	Deleterious	Disease causing
41–50	17					*TTN*	c.10966G>A	p.A3656T	rs72648923	0.3282/0.0267/0.2339	0.3419	—	VUS	—	Damaging	Neutral	Disease causing
41–50	18	NGS	45	H	HCM	*TTN*	c.62584G>A	p.V20862I	rs549709481	—	0.002488	—	VUS	—	Damaging	Neutral	Disease causing
41–50	18					*TTN*	c.289G>A	p.V97M	rs185921345	0.0349/0.0227/0.0308	0.2084	—	VUS	—	Damaging	Neutral	Disease causing
41–50	19	NGS	46	M	DCM	*TTN*	c.23131A>G	p.I7711V	rs72648994	0.0486/0.0524/0.0498	0.43	—	VUS	—	Damaging	Neutral	Polymorphism
41–50	19					*TTN*	c.67191A>C	p.Q22397H	rs201512527	0.1936/0.0262/0.1406	0.09869	—	VUS	—	Damaging	Neutral	Polymorphism
41–50	19					*TTN*	c.95443G>C	p.E31815Q	rs148525155	0.0603/0.512/0.0574	0.3337	—	VUS	—	Benign	Deleterious	Disease causing
41–50	20	NGS	46	M	HCM	*TTN*	c.96220-96222delCCT	p.P32074del	—	—	—	—	PPV	—	—	Deleterious	Disease causing
41–50	21	NGS	46	M	HCM	*RYR2*	c.649A>G	p.I217V	rs200642525	0.0362/0.0/0.0246	0.01408	CM125874	PPV	LQTS	Benign	Neutral	Disease causing
41–50	21					*TTN*	c.61160G>C	p.G20387A	rs201381085	0.0366/0.0/0.0252	0.02398	—	VUS	—	Damaging	Deleterious	Disease causing
41–50	22	NGS	47	F	DCM	*ANK2*	c.2048A>G	p.D683G	—	—	—	—	VUS	—	Benign	Neutral	Polymorphism
41–50	22					*RYR2*	c.3380A>G	p.E1127G	rs200525962	0.0832/0.0/0.0556	—	—	VUS	—	Damaging	Deleterious	Disease causing
41–50	22					*DMD*	c.8555A>G	p.K2852R	—	—	0.001181	—	VUS	—	Benign	Neutral	Disease causing
41–50	22					*VCL*	c.1907A>G	p.H636R	rs71579374	0.1279/0.0227/0.0923	0.1492	—	VUS	—	Damaging	Deleterious	Disease causing
41–50	23	NGS	48	M	HCM	*TTN*	c.30760-30762delGAA	p.E10254del	—	—	—	—	PPV	—	—	Deleterious	Polymorphism
41–50	24	NGS	49	M	HCM	*PKP2*	c.1468C>T	p.R490W	rs149930872	0.0233/0.159/0.0693	0.06146	—	VUS	—	Damaging	Neutral	Polymorphism
41–50	25	NGS	49	F	HCM	*PKP2*	c.712G>T	p.G269R	—	—	0.0008264	—	VUS	—	Damaging	Neutral	Disease causing
41–50	25					*CACNA1C*	c.805G>A	p.N550S	—	—	0.003790	—	PBV	—	Benign	Neutral	Polymorphism
41–50	25					*TTN*	c.55862G>T	p.G18621V	—	—	—	—	PPV	—	Damaging	Deleterious	Disease causing
41–50	26	NGS	49	M	DCM	*ANK2*	c.158G>C	p.G53A	—	—	0.0008860	—	VUS	—	Damaging	Deleterious	Polymorphism
41–50	26					*TTN*	c.89737G>A	p.G29913R	rs111616037	0.0119/0.0246/0.016	0.003876	—	VUS	—	Damaging	Deleterious	Disease causing

NGS, Next Generation sequencing. Age is expressed in years, months (m) or days (d). Autopsy is expressed as Hypertrophy Cardiomyopathy (HCM), Dilated Cardiomyopathy (DCM) and Fibrosis. Variant nomenclature is at cDNA and Protein level. Minor Allele Frequency (MAF) is expressed as EA/AA/ALL respectively, EA (European-American)/AA (African-American)/ALL; and Minor Alelle Frequency from The Exome Aggregation Consortium (ExAC), both are expressed in percentage. Each variant is classified as Disease Mutation (DM), Probably Pathogenic Variant (PPV), Variant of Uncertain Significance (VUS) and Probably Benign Variant (PBV).

### Unexplained sudden death subgroup

The analysis was performed in 119 samples with negative results after macroscopic and histological studies.

All samples were analyzed using NGS technology except in 24 samples in which the screening was limited to Sanger sequencing due to low DNA quality. The Sanger sequencing method identified at least one rare variant in 6 out of 24 (25%) samples. On the other hand, with the use of NGS technology we identified 76 out of 95 (80%) samples carrying at least one rare variant. Concerning NGS samples, 41 of them (43.2%) carried at least one rare variant in genes encoding proteins associated with ion channels, and 64 (67.3%) carried at least one rare variant in genes codifying for structural proteins. Overall, we detected 49(41.2%) samples carrying PPV and/or DM. The genetic screening identified a total of 197 rare variants, 6 detected by Sanger method and 191 by NGS method. Thirty-six (18.27%) were novel variants. Our criteria classified 16 (8.12%) as PBV, 100 (50.76%) as VUS, 60 (30.46%) as PPV and 21 (10.66%) as DM (Table 2).

**Table 2:** Genetic data of variants identified, 81 positive cases from the 119 SUD cases.

Range	ID	Proc.	Age	Gender	Autopsy	Gene	Nucleotide	Variant	dbSNP	MAF (%)	ExAC %	HGMD	Classification	Disease	PPH2	Provean	Mut. Taster
0–10	1	S	1m	F	SIDS	*SCN5A*	c.5054A>T	p.E1685D	—	—	—	BM1492175	PPV	SCD	Damaging	Deleterious	Disease causing
0–10	1					*SCN5A*	c.5055G>T	p.V1685V	—	—	—	CM1413453	PPV	SCD	Damaging	Deleterious	Disease causing
0–10	2	NGS	3d	M	SIDS	*TTN*	c.74377C>G	p.P24793A	rs56137800	—	0.2859	—	VUS	—	Damaging	Deleterious	Disease causing
0–10	2					*TTN*	c.11446G>A	p.V3816I	rs72648929	—	0.2733	—	VUS	—	Damaging	Neutral	Disease causing
0–10	3	NGS	13d	F	SIDS	*DSP*	c.5598G>C	p.Q1866H	—	—	0.0008238	—	PBV	—	Benign	Neutral	Polymorphism
0–10	3					*TTN*	c.74195G>A	p.R24732H	rs55850344	0.0608/0.0528/0.0583	0.03066	—	PBV	—	Benign	Neutral	Polymorphism
0–10	4	NGS	1m	F	SIDS	*SCN5A*	c.5848G>T	p.V1950L	rs41315493	0.0596/0.457/0.191	0.005412	CM024652	PPV	BrS	Benign	Neutral	Polymorphism
0–10	4					*DSG2*	c.1003A>G	p.T335A	rs191564916	0.061/0.0/0.0419	0.05558	CM098196	PPV	ARVC	Damaging	Neutral	Polymorphism
0–10	5	NGS	41d	M	SIDS	*MYH6*	c.595G>A	p.A199T	rs535526291	—	—	—	PPV	—	Damaging	Deleterious	Disease causing
0–10	5					*TTN*	c.16752C>G	p.I5584M	rs563320328	—	0.001668	—	PBV	—	Benign	Neutral	Polymorphism
0–10	6	NGS	2m	M	SIDS	*CACNA1C*	c.5809C>T	p.R1937C	rs185788586	0.1437/0.1257/0.1379	0.7488	CM1413436	PPV	SCD	Benign	Neutral	Disease causing
0–10	7	NGS	2m	F	SIDS	*SCN5A*	c.1844G>A	p.G615E	rs12720452	0.0595/0.0/0.0399	0.023	CM022061	PPV	LQTS-DA	Damaging	Neutral	Polymorphism
0–10	8	NGS	3m	M	SIDS	*TTN*	c.81017G>A	p.R27006H	rs111727915	0.0239/0.1711/0.0722	0.04143	—	VUS	—	Damaging	Deleterious	Disease causing
0–10	8					*TTN*	c.70109G>C	p.W23370S	rs186681106	0.0244/0.3203/0.1171	0.05575	—	VUS	—	Damaging	Deleterious	Disease causing
0–10	8					*TTN*	c.64675G>A	p.E21559K	rs149763294	0.0243/0.1318/0.0582	0.03929	—	VUS	—	Damaging	Deleterious	Disease causing
0–10	8					*TTN*	c.28025C>A	p.P9342Q	rs200459347	0.0243/0.1326/0.0583	0.04174	—	VUS	—	Benign	Deleterious	Polymorphism
0–10	8					*TTN*	c.18047C>A	p.S6016Y	rs187925021	0.0245/0.1609/0.0672	0.449	—	VUS	—	Benign	Deleterious	Polymorphism
0–10	8					*TTN*	c.17936G>A	p.R5979H	rs138853909	0.0362/0.155/0.074	0.05243	—	PBV	—	Benign	Neutral	Polymorphism
0–10	8					*TTN*	c.17600T>C	p.M5867T	rs374408615	0.012/0.0/0.0081	0.00161	—	PBV	—	Benign	Neutral	Polymorphism
0–10	9	NGS	1	F	SIDS	*DMD*	c.4529A>G	p.K1510R	rs72468638	0.0297/0.0/0.0189	0.5399	—	PBV	—	Damaging	Neutral	Polymorphism
0–10	9					*PRKAG2*	c.1107-5C>T	—	—	—	—	—	VUS	—	—	—	—
0–10	10	NGS	14m	M	U	*JUP*	c.283G>C	p.G95R	—	—	—	—	VUS	—	Benign	Neutral	Polymorphism
0–10	11	NGS	16m	M	U	*FBN1*	c.8176C>T	p.R2726W	rs61746008	0.1047/0.1137/0.1078	0.07331	CM950453	PPV	Marfan	Benign	Deleterious	Disease causing
0–10	11					*TNNC1*	c.304C>A	p.R102S	—	—	—	—	VUS	—	Benign	Deleterious	Disease causing
0–10	11					*GPD1L*	c.520G>A	p.E174K	rs112122950	0.1279/0.0227/0.0923	0.01483	—	VUS	—	Benign	Neutral	Disease causing
0–10	11					*TTN*	c.44832C>G	p.N14944K	rs199615557	0.01279/0/0.0333	0.03031	—	VUS	—	Benign	Deleterious	Disease causing
0–10	12	NGS	18m	F	U	*CACNA1C*	c.6062G>A	p.R2021Q	rs112414325	0.2183/0.0518/0.1652	0.3594	CM1413437	PPV	SCD	Bening	Neutral	Disease causing
0–10	12					*DSP*	c.916G>A	p.A306T	rs368193211	0.0116/0.0/0.0077	0.004944	CM1413441	PPV	SCD	Damaging	Neutral	Disease causing
0–10	13	NGS	3	M	U	*DSP*	c.314G>A	p.R105Q	—	—	0.004118	CM1413443	PPV	SCD	Damaging	Neutral	Disease causing
0–10	13					*DSP*	c.946A>G	p.M316V	rs201672777	0.0116/0/0.0077	0.002484	CM1413442	PPV	SCD	Damaging	Deleterious	Disease causing
0–10	13					*CACNB2*	c.47_49delCGG	p.A16fs	—	—	—	—	PPV	—	—	—	Disease causing
0–10	13					*DMD*	c.7183G>A	p.A2395T	rs72466590	0.2229/0/0.142	0.07408	CM072994	DM	MD	Benign	Neutral	Polymorphism
0–10	13					*FBN1*	c.1175C>G	p.P392R	rs534127494	—	0.003345	CM1413444	PPV	SCD	Damaging	Deleterious	Disease causing
0–10	14	NGS	3	M	SUDEP	*TTN*	c.89494C>A	p.P29832T	rs373876117	0.0122/0.0/0.0084	0.01195	—	VUS	—	Damaging	Deleterious	Disease causing
0–10	14					*TTN*	c.5577G>T	p.R1859S	—	—	0.003295	BM1437281	PPV	SCD	Damaging	Deleterious	Polymorphism
0–10	15	NGS	3	M	U	*TTN*	c.77076A>C	p.E25693D	—	—	0.002485	—	VUS	—	Benign	Neutral	Disease causing
0–10	16	S	10	M	U	*KCNH2*	c.2674C>T	p.R892C	rs201627778	0.0116/0.0227/0.0154	0.04794	CM1413446	PPV	SCD	Damaging	Deleterious	Disease causing
11–20	17	NGS	14	F	U	*DSC2*	c.1789G>T	p.V597F	rs143040393	0.0116/0.2497/0.0923	0.01818	CM1413440	PPV	SCD	Benign	Deleterious	Polymorphism
11–20	17					*CACNA1C*	c.5809C>T	p.R1937C	rs185788586	0.1437/0.1257/0.1379	0.7488	CM1413436	PPV	SCD	Damaging	Neutral	Disease causing
11–20	17					*ANK2*	c.7148C>T	p.P2383L	rs35960628	0/0.0908/0.0308	0.01237	CM1413435	PPV	SCD	Benign	Neutral	Polymorphism
11–20	17					*TGFB3*	c.755-5T>C	—	—	—	—	—	VUS	—	—	—	—
11–20	17					*DMD*	c.4328A>G	p.Q1443R	—	—	0.001150	CM1413439	PPV	SCD	Damaging	Neutral	Disease causing
11–20	18	NGS	19	M	U	*TTN*	c.27470A>G	p.Y9157S	—	—	—	—	VUS	—	Benign	Neutral	Polymorphism
11–20	19	NGS	20	M	U	*ANK2*	c.4373A>G	p.E1458G	rs72544141	0.0233/0/0.0154	0.04222	CM030186	DM	LQTS	Damaging	Deleterious	Disease causing
11–20	19					*TNNT2*	c.832C>T	p.R278C	rs121964857	0.0582/0.0227/0.461	0.04291	CM951222	DM	HCM	Damaging	Neutral	Disease causing
11–20	19					*TTN*	c.64001T>C	p.I21334T	rs55837610	0.3873/0.026/0.2725	0.2155	—	VUS	—	Benign	Deleterious	Polymorphism
11–20	20	NGS	20	M	U	*CACNA1C*	c.5086G>A	p.A1696T	rs370432385	0.0119/0.0/0.008	0.008678	—	VUS	—	Benign	Neutral	Disease causing
21–30	21	NGS	21	M	SUDEP	*FBN1*	c.5443G>A	p.G1815S	—	—	0.001649	—	VUS	—	Damaging	Neutral	Disease causing
21–30	21					*HCN4*	c.2452G>A	p.G818S	—	—	—	—	VUS	—	Damaging	Neutral	Disease causing
21–30	22	NGS	22	M	U	*VCL*	c.510C>T	p.T197I	rs189242810	0.0349/0.0/0.0231	0.02307	—	VUS	—	Benign	Neutral	Disease causing
21–30	23	NGS	22	H	U	*MYH6*	c.3428G>A	p.R1143Q	rs543585784	—	0.0111	—	VUS	—	Damaging	Neutral	Disease causing
21–30	23					*TPM1*	c.451G>A	p.A151T	—	—	—	—	VUS	—	Damaging	Neutral	Disease causing
21–30	23					*TTN*	c.74315T>A	p.I24772K	rs371592971	0.0122/0.0/0.0084	0.002485	—	VUS	—	Damaging	Neutral	Disease causing
21–30	24	NGS	23	F	U	*TTN*	c.98971G>C	p.E32991Q	rs199632397	0.0245/0.0275/0.0254	0.04170	—	VUS	—	Damaging	Neutral	Polymorphism
21–30	24					*TTN*	c.5993G>A	p.R1998H	rs144135510	0.1163/0/0.0769	0.1756	—	VUS	—	Damaging	Deleterious	Disease causing
21–30	24					*TTN*	c.74366C>G	p.T24789R	—	—	0.002486	—	VUS	—	Damaging	Deleterious	Disease causing
21–30	25	NGS	23	M	U	*KCNQ1*	c.1343C>G	p.P448R	rs12720449	0.0116/0.0/0.0077	0.7614	CM002332	PPV	LQTS	Benign	Neutral	Polymorphism
21–30	25					*TTN*	c.87857G>C	p.W29286S	—	—	—	—	PPV	—	Damaging	Deleterious	Disease causing
21–30	25					*TTN*	c.74842T>A	p.W24948R	—	—	0.0008286	—	VUS	—	Damaging	Deleterious	Disease causing
21–30	26	NGS	23	D	SUDEP	*JUP*	c.526C>T	p.R176W	rs368336007	0.0116/0.0/0.0077	0.006811	—	VUS	—	Damaging	Deleterious	Disease causing
21–30	26					*KCNH2*	c.1810G>A	p.G604S	rs199473522	—	—	CM990760	DM	LQTS	Damaging	Deleterious	Disease causing
21–30	27	NGS	24	M	U	*RYR2*	c.2047G>A	p.E683K	—	—	—	—	PPV	—	Damaging	Neutral	Disease causing
21–30	27					*TTN*	c.29295T>A	p.Y9766stop	—	—	—	—	PPV	—	—	Deleterious	Disease causing
21–30	28		24	M	U	*SCN5A*	c.393-5C>A	—	rs368678204	0.0118/0.0/0.0078	0.01297	CS097852	PPV	LQTS	—	—	—
21–30	28					*FBN1*	c.698G>A	p.R233H	rs770140872	—	0.002477	—	VUS	—	Damaging	Deleterious	Disease causing
21–30	28					*TTN*	c.2776-4C>A	—	—	—	0.0008331	—	PPV	—	—	—	—
21–30	29	NGS	24	F	U	*KCNH2*	c.1757T>C	p.L586P	—	—	—	—	PPV	—	Damaging	Deleterious	Disease causing
21–30	29					*TNNC1*	c.337G>A	p.D113N	rs369639550	0./00227/0.0077	—	—	VUS	—	Damaging	Neutral	Disease causing
21–30	29					*TTN*	c.79708C>A	p.P26570T	rs72648227	0.0121/0/0.0083	0.1093	—	VUS	—	Damaging	Deleterious	Disease causing
21–30	29					*TTN*	c.39487C>T	p.R13163C	rs72677231	0.2887/0.517/0.2136	0.2249	—	VUS	—	Damaging	Deleterious	Disease causing
21–30	29					*TTN*	c.8884G>A	p.A2962T	rs376039623	0.0243/0.0/0.0167	0.0008986	—	VUS	—	Damaging	Deleterious	Disease causing
21–30	30	NGS	24	F	U	*RYR2*	c.1392C>A	p.H464Q	—	—	—	—	VUS	—	Benign	Neutral	Disease causing
21–30	30					*DSG2*	c.1912G>A	p.G638R	rs201564919	0.0242/0/0.0165	0.01244	CM109865	DM	ARVC	Damaging	Deleterious	Disease causing
21–30	30					*TTN*	c.84472C>T	p.P28158S	rs72648247	0.2305/0.0524/0.1742	0.2484	—	VUS	—	Damaging	Deleterious	Disease causing
21–30	30					*TTN*	c.29781_29783dupAGA	p.E9928dup	rs368327166	—	—	—	PPV	—	—	—	Disease causing
21–30	31	NGS	24	M	U	*PKP2*	c.1592T>G	p.I531S	rs147240502	0.4884/0.0/0.3229	0.4722	CM102861	PPV	ARVC	Damaging	Deleterious	Disease causing
21–30	31					*HCN4*	c.3082C>G	p.P1028A	—	—	—	—	VUS	—	Benign	Neutral	Polymorphism
21–30	31					*TTN*	c.85866T>A	p.N28623K	—	—	0.004143	—	VUS	—	Damaging	Deleterious	Disease causing
21–30	31					*TTN*	c.79896G>C	p.M26633I	—	—	0.02733	—	VUS	—	Damaging	Neutral	Disease causing
21–30	31					*TTN*	c.43823G>C	p.G14608A	—	—	0.02735	—	VUS	—	Damaging	Deleterious	Disease causing
21–30	32	NGS	27	M	U	*SCN4B*	c.613T>C	p.S205P	—	—	—	—	PPV	—	Damaging	Deleterious	Disease causing
21–30	33	NGS	29	M	U	*HCN4*	c.2938G>A	p.G980R	—	—	0.003473	—	VUS	—	Damaging	Neutral	Polymorphism
21–30	33					*ANK2*	c.2938G>A	p.A2948Q	rs138438183	0.0349/0.0227/0.0308	0.01898	—	VUS	—	Benign	Neutral	Polymorphism
21–30	33					*TTN*	c.68678T>C	p.I22893T	—	—	0.0008334	—	VUS	—	Damaging	Neutral	Disease causing
21–30	34	NGS	30	M	U	*VCL*	c.829C>A	p.L277M	rs71579353	0.0116/0.0/0.0077	0.004126	CM062022	DM	HCM	Benign	Neutral	Disease causing
21–30	34					*TTN*	c.31720C>T	p.P10574S	rs200992277	—	—	—	VUS	—	Benign	Deleterious	Polymorphism
21–30	35	NGS	30	M	U	*ANK2*	c.4912A>G	p.N1638D	—	—	—	—	VUS	—	Benign	Neutral	Polymorphism
21–30	35					*TTN*	c.88582G>A	p.A29528T	rs376039623	0.0243/0.0/0.0167	0.006650	—	VUS	—	Benign	Neutral	Disease causing
21–30	36	NGS	30	M	U	*CASQ2*	c.730C>T	p.H244Y	rs142036299	—	0.02327	—	VUS	—	Damaging	Deleterious	Disease causing
31–40	37	NGS	33	F	U	*DES*	c.935A>C	p.D312A	rs148947510	0.0/0.2951/0.1	0.03891	CM137784	PPV	CP	Damaging	Deleterious	Disease causing
31–40	38	NGS	33	M	U	*MYH7*	c.4879A>T	p.I1627F	—	—	—	—	VUS	—	Benign	Neutral	Disease causing
31–40	38					*TTN*	c.89786T>C	p.I29929T	rs55660660	0.0119/0.5682/0.1932	0.09549	—	VUS	—	Damaging	Deleterious	Disease causing
31–40	38					*TTN*	c.81004A>G	p.I27002V	rs139506970	0.0119/0.636/0.2166	0.07539	—	VUS	—	Benign	Neutral	Disease causing
31–40	38					*TTN*	c.76036A>G	p.G19020R	rs181717727	0.024/0.6021/0.211	0.08071	—	VUS	—	Damaging	Deleterious	Polymorphism
31–40	38					*TTN*	c.57058G>A	p.R922H	rs56046320	0.0116/0.9305/0.3229	0.09066	—	VUS	—	Benign	Neutral	Disease causing
31–40	38					*TTN*	c.2765G>A	p.T25346A	rs188370772	0.0121/0.6282/0.2053	0.07464	—	VUS	—	Benign	Deleterious	Polymorphism
31–40	39	NGS	33	F	U	*PKP2*	c.1872G>T	p.E624D	rs370219248	0.0/0.0227/0.0077	0.006593	—	VUS	—	Benign	Neutral	Polymorphism
31–40	39					*ANK2*	c.8768A>G	p.Q2923R	rs551454026	—	0.09067	—	VUS	—	Benign	Neutral	Polymorphism
31–40	39					*TTN*	c.30515_17delAAG	p.E10172fs	rs397517549	—	—	—	PPV	—	—	—	Disease causing
31–40	40	NGS	34	M	U	*RYR2*	c.8145G>T	p.E2715D	rs200420897	0.0126/0.0283/0.0175	—	—	VUS	—	Damaging	Deleterious	Disease causing
31–40	40					*TTN*	c.93961G>A	p.V33889I	rs34924609	0.6481/0.1623/0.4969	0.3311	—	VUS	—	Benign	Neutral	Disease causing
31–40	41	NGS	34	M	U	*TTN*	c.47109T>G	p.F15703L	rs370583314	0.0124/0.0/0.0086	0.004479	—	VUS	—	Benign	Deleterious	Polymorphism
31–40	41					*TTN*	c.52341A>C	p.E17447D	rs575796706	—	0.001662	—	VUS	—	Benign	Neutral	Disease causing
31–40	41					*TTN*	c.45509A>T	p.D15170V	—	—	0.004973	—	VUS	—	Damaging	Deleterious	Disease causing
31–40	41					*JUP*	c.1717G>T	p.D573Y	—	—	—	—	PPV	—	Damaging	Deleterious	Disease causing
31–40	42	NGS	34	F	U	*DSP*	c.4372C>G	p.R1458G	rs28763965	0.2093/0.0908/0.1692	0.1737	CM113816	PPV	ARVC	Damaging	Neutral	Polymorphism
31–40	42					*CACNB2*	c.1180G>A	p.V394I	rs149793143	0.0/0.0227/0.0077	0.001649	CM127056	DM	BrS	Damaging	Neutral	Disease causing
31–40	42					*SCN5A*	c.6007T>C	p.F2004L	rs41311117	0.3107/0.0497/0.2259	0.2018	CM086913	PPV	BrS	Damaging	Neutral	Polymorphism
31–40	43	NGS	35	H	U	*MYBPC3*	c.3569G>T	p.R1190L	rs117354682	0.0/0.0254/0.0082	0.005870	—	VUS	—	Damaging	Deleterious	Disease causing
31–40	43					*MYBPC3*	c.961G>A	p.V321M	rs200119454	0.0471/0.0232/0.039	0.04625	CM115891	DM	DCM	Damaging	Neutral	Disease causing
31–40	44	S	36	M	U	*SCN5A*	c.3530C>G	p.P1177R	—	—	—	—	PPV	—	Damaging	Deleterious	Disease causing
31–40	45	NGS	36	F	U	*KCNJ2*	c.1229A>G	p.N410S	rs141069645	0.0233/0.0454/0.0308	0.03722	CM1313311	DM	LQTS	Benign	Neutral	Disease causing
31–40	46	NGS	36	F	U	*PKP2*	c.611G>A	p.R204H	rs755215178	—	0.007414	—	PBV	—	Benign	Neutral	Polymorphism
31–40	46					*FBN1*	c.83A>G	p.N28S	rs193922245	—	0.00659	—	PBV	—	Benign	Neutral	Polymorphism
31–40	47	NGS	37	M	U	*SCN5A*	c.4G>A	p.A2T	rs199473042	—	0.002537	CM104269	DM	BrS	Damaging	Neutral	Disease causing
31–40	47					*SCN5A*	c.1855C>T	p.L619F	rs199473133	0.0238/0.0/0.016	0.003699	CM030952	DM	LQTS	Damaging	Neutral	Disease causing
31–40	48	NGS	37	M	U	*JPH2*	c.1625G>A	p.R542H	rs369279135	0.0/0.0304/0.0096	0.005589	—	PBV	—	Benign	Neutral	Polymorphism
31–40	49	NGS	38	M	U	*PRKAG2*	c.298G>A	p.G100S	rs79474211	0.0814/0.0908/0.0846	0.8132	CM136115	DM	PRKAG2 syndrome	Damaging	Neutral	Disease causing
31–40	49					*ACTN2*	c.1975-6C>G	—	rs201255023	—	0.1120	—	VUS	—	—	—	—
31–40	49					*TTN*	c.77702C>G	p.S25901C	rs202040332	0.7558/0.227/0.5767	0.1666	—	VUS	—	Damaging	Deleterious	Disease causing
31–40	49					*FBN1*	c.6987C>G	p.D2329E	rs363831	0.0/0.1137/0.0385	0.06105	—	VUS	—	Neutral	Neutral	Polymorphism
31–40	49					*FBN1*	5672-3T>C	—	rs193922217	—	—	—	PPV	—	—	—	—
31–40	50	NGS	38	F	U	*DSP*	c.8402G>A	p.R2801H	—	—	0.0008249	—	PPV	—	Damaging	Deleterious	Disease causing
31–40	50					*TTN*	c.10503G>C	p.K3501N	—	—	—	—	VUS	—	Damaging	Neutral	Polymorphism
31–40	51	NGS	38	M	U	*SCN5A*	c.4648G>C	p.D1550H	—	—	—	—	PPV	—	Damaging	Deleterious	Disease causing
31–40	51					*TTN*	c.5419C>A	p.P1807T	rs200563229	—	0.0008254	—	VUS	—	Damaging	Deleterious	Disease causing
31–40	52	NGS	38	M	U	*DSP*	c.3399C>G	p.D1133E	—	—	—	—	PPV	—	Damaging	Deleterious	Disease causing
31–40	52					*DSC2*	c.2603C>T	p.S868F	rs141873745	0.0/0.0227/0.0077	0.005769	—	PBV	—	Benign	Neutral	Polymorphism
31–40	53	NGS	39	M	U	*TGFB3*	c.97G>A	p.G33S	—	—	0.003295	—	PBV	—	Benign	Neutral	Polymorphism
31–40	53					*TTN*	c.19013C>G	p.S6338C	—	—	0.003320	—	VUS	—	Damaging	Deleterious	Polymorphism
31–40	54	NSG	39	M	U	*TTN*	c.23200G>C	p.D7734H	—	—	0.0008503	—	VUS	—	Damaging	Deleterious	Disease causing
31–40	55	S	40	M	U	*KCNH2*	c.2119T>C	p.Y707H	—	—	—	—	PPV	—	Damaging	Deleterious	Disease causing
31–40	56	NGS	40	F	U	*FBN1*	c.4163G>A	p.R1388H	—	—	0.002477	—	VUS	—	Damaging	Deleterious	Disease causing
41–50	57	NGS	42	M	U	*MYH7*	c.5669A>G	p.N1890S	—	—	—	—	PPV	—	Damaging	Deleterious	Disease causing
41–50	57					*MYH7*	c.3235C>T	p.R1079W	rs192722540	—	0.004944	—	PPV	—	Damaging	Deleterious	Disease causing
41–50	57					*TTN*	c.21088G>A	p.E7030K	rs72648981	0.3747/0.0261/0.2642	0.198	—	VUS	—	Damaging	Deleterious	Disease causing
41–50	57					*TTN*	c.583+5G>A	—	—	—	—	—	VUS	—	—	—	—
41–50	58	NGS	42	M	U	*KCNJ2*	c.1229A>G	p.N410S	rs141069645	0.0233/0.0454/0.0308	0.03722	CM1313311	DM	LQTS	Benign	Neutral	Disease causing
41–50	59	NGS	42	M	U	*CACNB2*	c.209G>A	p.R70H	rs150722502	—	0.08816	—	PPV	—	Damaging	Deleterious	Disease causing
41–50	60	S	43	M	U	*RYR2*	c.12919C>T	p.R4307C	rs200092869	0.1092/0.0/0.0745	—	—	VUS	—	Damaging	Deleterious	Polymorphism
41–50	61	NGS	43	F	U	*KCNH2*	c.2860C>T	p.R954C	rs141401803	—	0.008263	CM070176	DM	SIDS	Damaging	Neutral	Disease causing
41–50	62	NGS	43	M	U	*CACNA1C*	c.6169C>T	p.R2057W	—	—	—	—	PPV	—	Damaging	Deleterious	Disease causing
41–50	62					*DMD*	c.6275A>G	p.Y2092C	rs745717858	—	0.001141	—	PPV	—	Damaging	Deleterious	Disease causing
41–50	62					*CASQ2*	c.928G>A	p.D310N	rs141314684	0.0581/0.0227/0.0461	0.06344	—	VUS	—	Benign	Deleterious	Disease causing
41–50	62					*PRKAG2*	c.1387G>T	p.V463L	—	—	—	—	PPV	—	Damaging	Deleterious	Disease causing
41–50	62					*RYR2*	c.4465T>C	p.1489R	rs200450676	0.0119/0.0/0.008	0.0166	—	VUS	—	Damaging	Deleterious	Disease causing
41–50	62					*KCNE2*	c.22A>G	p.T8A	rs2234916	0.686/0.1135/0.4921	0.3804	CM003449	VUS	LQTS-DA	Damaging	Deleterious	Disease causing
41–50	63	NGS	43	M	U	*MYBPC3*	c.3384G>C	p.E1128D	rs375116558	0.0241/0.0/0.0162	0.0127	—	VUS	—	Benign	Neutral	Disease causing
41–50	64	NGS	44	M	U	*KCNH2*	c.2941A>G	p.S981G	rs76649554	0.0116/0.0227/0.0154	0.04304	—	VUS	—	Benign	Neutral	Disease causing
41–50	65	NGS	44	M	U	*HCN4*	c.2210A>G	p.Q737R	rs146732972	0.0/0.1365/0.0462	0.02074	—	VUS	—	Damaging	Neutral	Disease causing
41–50	65					*PKP2*	c.1637C>A	p.A546E	—	—	—	—	VUS	—	Benign	Neutral	Disease causing
41–50	65					*CACNA1C*	c.667G>A	p.A223T	—	—	—	—	PPV	—	Damaging	Neutral	Disease causing
41–50	66	NGS	44	M	U	*ANK2*	c.11465G>C	p.G3822A	rs79577190	0.0/0.6809/0.2307	0.06609	—	PBV	—	Benign	Neutral	Polymorphism
41–50	66					*ANK2*	c.11791G>A	p.E3931K	rs45454496	0.4186/0.0908/0.3076	0.267	CM041240	DM	CA	Damaging	Neutral	Polymorphism
41–50	66					*TTN*	c.84206T>C	p.M28069T	—	—	—	—	VUS	—	Benign	Deleterious	Polymorphism
41–50	67	NGS	44	M	U	*MYH6*	c.3612G>C	p.E1204D	rs751153777	—	—	—	VUS	—	Benign	Neutral	Disease causing
41–50	67					*TTN*	c.50144-4G>A	—	rs369462016	0.0/0.0274/0.0085	0.002627	—	PPV	—	—	—	—
41–50	68	NGS	45	M	U	*JUP*	c.475G>T	p.V159L	—	—	0.004237	CM1010258	PPV	ARVC	Damaging	Deleterious	Disease causing
41–50	68					*KCNE3*	c.46G>A	p.A16T	—	—	0.003299	—	PBV	—	Benign	Neutral	Polymorphism
41–50	68					*KCNE3*	c.40C>A	p.L14M	—	—	0.003299	—	PPV	—	Damaging	Neutral	Disease causing
41–50	68					*TTN*	c.18248C>T	p.T6083M	—	—	0.01457	—	PBV	—	Benign	Neutral	Polymorphism
41–50	69	NGS	45	M	U	*MYBPC3*	c.1786G>A	p.G596R	rs199728019	0.0/0.0238/0.0079	0.02622	—	VUS	—	Damaging	Deleterious	Disease causing
41–50	70	NGS	46	M	U	*TTN*	c.55460C>T	p.P18487L	rs779343098	—	0.001820	—	VUS	—	Damaging	Deleterious	Disease causing
41–50	71	NGS	46	M	U	*DSP*	c.1140+6T>C	—	rs534740669	—	—	—	VUS	—	—	—	—
41–50	71					*TTN*	c.40796G>A	p.R13599Q	—	—	0.0008291	—	VUS	—	Damaging	Neutral	Polymorphism
41–50	72	NGS	47	M	U	*CACNA1C*	c.6029G>A	p.R2010Q	rs199776761	0.0122/0.0522/0.0249	0.01333	—	PBV	—	Benign	Neutral	Polymorphism
41–50	72					*DES*	c.635G>A	p.R212Q	rs144261171	—	0.02059	—	VUS	—	Damaging	Deleterious	Disease causing
41–50	72					*TTN*	c.92737A>G	p.I30913V	—	—	—	—	VUS	—	Benign	Neutral	Polymorphism
41–50	72					*TTN*	c.6950G>A	p.R2317H	—	—	0.004945	—	VUS	—	Damaging	Deleterious	Disease causing
41–50	73	NGS	48	M	U	*SCN5A*	c.1440A>C	p.K480N	—	—	0.0008383	—	PPV	—	Damaging	Deleterious	Disease causing
41–50	73					*TTN*	c.60754G>C	p.A20252P	rs72646880	0.315/0.0264/0.2242	0.1961	CM1413461	PPV	SCD	Damaging	Deleterious	Disease causing
41–50	73					*TTN*	c.52846G>A	p.V17616I	rs564621227	—	0.002489	—	VUS	—	Benign	Neutral	Disease causing
41–50	74	NGS	48	F	U	*MYH7*	c.3613G>A	p.E1205K	—	—	0.0008536	CM081343	DM	HCM	Damaging	Deleterious	Disease causing
41–50	75	NGS	48	M	U	*VCL*	c.1907A>G	p.H636R	rs71579374	0.1279/0.0227/0.0923	0.1492	—	VUS	—	Damaging	Deleterious	Disease causing
41–50	75					*TTN*	c.68360A>G	p.H22787R	—	—	—	—	VUS	—	Benign	Deleterious	Disease causing
41–50	76	NGS	48	M	U	*FBN1*	c.3463+3A>G	—	rs80344206	0.0/0.0227/0.0077	0.3114	—	VUS	—	—	—	—
41–50	76					*LDB3*	c.163G>A	p.V55I	rs3740343	0.1047/0.1135/0.1076	0.7222	—	VUS	—	Benign	Neutral	Disease causing
41–50	76					*TTN*	c.99430A>C	p.N33144H	—	—	0.009807	—	VUS	—	Damaging	Deleterious	Disease causing
41–50	76					*TTN*	c.86021G>A	p.R28674H	rs369899675	0.012/0.0/0.0081	0.01079	—	VUS	—	Damaging	Deleterious	Disease causing
41–50	77	NGS	48	M	U	*DSG2*	c.1003A>G	p.T335A	rs191564916	0.061/0.0/0.0419	0.05558	CM098196	PPV	ARVC	Damaging	Neutral	Polymorphism
41–50	77					*SCN5A*	c.1140+2T>C	—	—	—	—	—	PPV	—	—	—	—
41–50	78		49	M	U	*CAV3*	c.216C>G	p.C72W	rs116840776	0.1744/0.0681/0.1384	0.1125	CM980306	DM	MD	Damaging	Deleterious	Disease causing
41–50	78					*ANK2*	c.7372A>T	p.S2458C	—	—	—	—	PPV	—	Damaging	Deleterious	Disease causing
41–50	78					*MYH6*	c.3010G>T	p.A1004S	s143978652	0.1279/0.0227/0.0923	0.09801	CM052257	DM	DCM	Benign	Neutral	Disease causing
41–50	78					*TGFBR2*	c.1159G>A	p.V387M	rs35766612	0.3023/0.0908/0.2307	0.1156	CM063201	DM	TAA	Damaging	Neutral	Disease causing
41–50	78					*LMNA*	c.1580G>A	p.R527H	rs57520892	0.0116/0.0/0.0077	0.006832	CM021630	DM	Mandibular dysplasia	Benign	Deleterious	Disease causing
41–50	79	NGS	49	M	U	*SCN5A*	c.1715C>A	p.A572D	rs36210423	0.2388/0.0245/0.1685	0.4304	CM034060	PPV	LQTS	Benign	Neutral	Polymorphism
41–50	79					*MYBPC3*	c.2497G>A	p.A833T	rs199865688	0.1758/0.0/0.1166	0.1682	CM032957	DM	HCM	Damaging	Neutral	Disease causing
41–50	79					*TTN*	c.58726G>A	p.A19576T	rs183276016	0.0242/0.0261/0.0248	0.01746	—	VUS	—	Damaging	Neutral	Disease causing
41–50	79					*TTN*	c.89494C>A	p.P29832T	rs373876117	0.0122/0.0/0.0084	0.01195	CM1413459	PPV	SCD	Damaging	Deleterious	Disease causing
41–50	79					*TTN*	c.17387G>T	p.R5796L	—	—	—	—	VUS	—	Damaging	Deleterious	Polymorphism
41–50	80	NGS	50	F	U	*MYH7*	c.3382G>A	p.A1128T	rs61741930	—	0.01147	—	VUS	—	Damaging	Neutral	Disease causing
41–50	80					*ANK2*	c.11725T>C	p.S3909P	rs141124755	0.0116/0.2724/0.1	0.03627	—	VUS	—	Benign	Neutral	Polymorphism
41–50	80					*TTN*	c.87872A>C	p.K29291T	—	—	—	—	VUS	—	Benign	Deleterious	Polymorphism
41–50	81	NGS	50	M	U	*TCAP*	c.171C>G	p.C57W	rs369447207	0.0116/0.0/0.0077	0.003123	—	VUS	—	Damaging	Deleterious	Disease causing
41–50	81					*TTN*	c.27913A>G	p.I9305V	rs376613199	0.0123/0.0/0.0085	0.02567	—	PBV	—	Benign	Neutral	Polymorphism
41–50	81					*TTN*	c.835C>T	p.R279W	rs138060032	0.0116/0.0/0.0077	0.01318	—	VUS	—	Damaging	Deleterious	Disease causing

NGS, Next Generation sequencing. Age is expressed in years, months (m) or days (d). Gender is expressed as Males (M) and Females (F). Autopsy is expressed as Unexplained (U), Sudden Infant Death Syndrome (SIDS) or Sudden Unexplained Death cases with Epilepsy (SUDEP). Variant nomenclature is at cDNA and Protein level. S means Sanger technology. SCD means Sudden Cardiac Death. Minor Allele Frequency (MAF) is expressed as EA/AA/ALL respectively, EA (European-American)/AA (African-American)/ALL; and Minor Alelle Frequency from The Exome Aggregation Consortium (ExAC), both are expressed in percentage. Each variant is classified as Disease Mutation (DM), Probably Pathogenic Variant (PPV), Variant of Uncertain Significance (VUS) and Probably Benign Variant (PBV). TAA means Thoracic Aortic Aneurysm. MD means Muscular Dystrophy. CA means Cardiac Arrhythmia. CP means Cardiomyopathy. LQTS-DA means Long QT Syndrome Drug-Associated.

Concerning ranges of age, the youngest cohort (between 0–10 years of age) included 28 samples (14 males -50%-, and 14 females -50%-). A total of 11 samples were screened by Sanger method, and 17 by NGS. The genetic screening by both technologies identified a total of 37 rare variants, 3 detected by Sanger method and 34 by NGS method. Four variants (10.81%) were novel. We detected 16 samples carrying at least one rare genetic variant (57.14%). Six samples (21.43%) carried variants in genes associated with ion channels and 12 samples (42.86%) carried variants in genes codifying for structural proteins. We identified 10 (35.71%) samples carrying PPV and/or DM. Finally, pathogenicity criteria classified 6 variants (16.22%) as PBV, 14 (37.84%) as VUS, 16 (43.24%) as PPV and 1 (2.7%) as DM.

The cohort between 11–21 years of age included 7 samples (5 males -71.43%-, and 2 females -28.57%-). A total of 2 samples were screened by Sanger method, 5 by NGS. The genetic screening identified a total of 10 rare variants by NGS method. The Sanger screening did not identify positive samples carrying rare variants. Of identified variants, 2 (20%) were novel. NGS method identified 4 samples (80%) carrying at least one rare variant, 3 (42.86%) carried variants in genes associated with ion channels, and 4 (57.14%) in genes codifying for structural proteins. Our classification criteria for all samples screened identified 2 (28.57%) samples carrying PPV and/or DM. Finally our classification criteria divided the 10 variants in 4 (40%) VUS, 4 (40%) PPV and 2 (20%) DM.

The cohort between 21–30 years of age included 19 samples (14 males -73.68%-, and 5 females -26.32%-). All were screened by NGS. The genetic screening identified a total of 42 rare variants. Of all them, 10 (23.81%) were novel. At least one rare variant was detected in 16 samples (84.21%), 12 (63.16%) in genes associated with ion channels, and 15 (78.95%) in genes codifying for structural proteins. Our criteria classified 9 (47.37%) as PPV and/or DM. Finally our classification criteria divided the 42 variants in 29 (69.05%) VUS, 10 (23.81%) PPV, and 3 (7.14%) DM.

The cohort between 31–40 years of age included 29 samples (20 males -68.97%-, and 9 females -31.03%-). A total of 3 samples were screened by Sanger method, 26 by NGS. Sanger screening identified 2 (66.67%) positive samples for rare variants. The genetic screening by both methods identified a total of 44 rare variants, 2 (4.5%) detected by Sanger method and 42 (95.5%) by NGS method. Of all them, 7 (15.91%) were novel. NGS method identified 18 samples (69.23%) carrying at least one rare variant, 6 (33.3%) carried variants in genes associated with ion channels, and 16 (55.2%) in genes codifying for structural proteins. Our classification criteria for all samples screened identified 13 (44.83%) samples carrying PPV and/or DM. Finally our criteria classified the 44 variants in 5 (11.36%) PBV, 22 (50%) VUS, 11 (25%) PPV and 6 (13.64%) DM.

The oldest cohort (between 41–50 years old), included 36 samples (28 males -77.78%-, and 8 females -22.22%-). A total of 8 samples were screened by Sanger method, 28 by NGS. The genetic screening by both methods identified a total of 64 rare variants, 1 (1.6%) detected by Sanger method and 63 (98.44%) by NGS method. Of all them, 13 (20.31%) were novel. NGS method identified 24 samples (85.7%) carrying at least one rare variant, 14 (50%) in genes associated with ion channels, and 20 (71.4%) in genes codifying for structural proteins. Our criteria classified 14 (38.89%) variants as PPV and/or DM. Finally our criteria classified the 64 variants in 5 (7.81%) PBV, 33 (51.56%) VUS, 17 (26.56%) PPV, and 9 (14.06%) DM.

## Discussion

In this prospective cohort we have methodically examined the etiology of natural death by performing a comprehensive investigation that includes a thorough autopsy examination and the inclusion of an extensive molecular autopsy. We have analyzed a total of 789 SD cases younger than 50 years of age. By concentrating all our cases in a same institution, a same autopsy protocol was followed, according to international forensic recommendations[1, 18].

Regarding epidemiological data, our data are in concordance with previous studies [32]. Thus we observed similar results in victims’ mean age (39.3 years old), gender (77.19% males), progressive increase in SD prevalence from age 0 to 50, and predominance of male deaths in all ranges of age.

The primary cause of SD was cardiac -81.1%-, similar to other cohorts. In 56.87% of cases, death was labeled from coronary artery disease (CAD), either from evidence of myocardial infarction or from the identification of severe coronary stenosis, which induced ischemia-related arrhythmias [28]. This percentage is not much different than the one reported in other studies, which attributed nearly 40% of deaths to ischemic heart disease[33]. However, in other reports the percentage of CAD neared 80%. The difference in our percentage is probably due to the inclusion of population only younger than 50 years of age in our cohort. The percentage of CAD-related death increased with age and was highest in population above 40 years old, as it was expected. In 2015, Vassalini et al reported the presence of ischaemic alterations in 18.5% of a cohort of patients aged less than 40 years, while other studies have underlined higher incidences of coronary related SCD, with percentages ranging between 48 and 73% [34–38]. These discrepancies can be explained by the different diagnostic criteria used in sample selection and age cut-off.

Regarding the situation of death, most CAD-related SD occurred in population older than 40 years of age, and during daily activity or stress/exercise. In the young population, most cardiomyopathy-related cases died during stress/exercise. These results are in concordance with other published cohorts and well-known data about exercise being a significant risk factor for cardiac death.

Macroscopic autopsy was able to define the cause of death in most cases above age 30. However, in cases below age 30, a negative macroscopic autopsy was the most common scenario. After adding histological analysis, an additional 17% of cases were labeled as of cardiac origin, cardiomyopathy or coronary artery disease, increasing the total percentage of cardiac origin to 46%, with CAD totaling 28.5%. In the young population, the main cause of death was from cardiomyopathies and in younger than 10 years of age, inherited cardiac diseases were the primary cause, also similar to published reports [39–42]. Nearly 19% of our cases remained as a negative autopsy, in concordance with a recent publication of Vassalini et al [43]. In other reports this percentage ranges from 5% to 40%, probably related to the study cohort as well as autopsy protocols [33, 44].

In 2015, the Swiss Society of Legal Medicine created a multidisciplinary working group, clinical and molecular geneticists together with cardiologists, in the hope of harmonizing the approach to the investigation of SCD. The key points of the recommendations were (1.) the realization of a forensic autopsy procedure for all SCD victims under 40 years of age (molecular autopsy or post-mortem genetic testing), (2.) the collection and storage of adequate samples for genetic testing, (3.) communication with the families, and (4.) a multidisciplinary approach including cardiac genetic counseling[19]. Though, despite these recommendations and the increasing availability of NGS technology, it is yet seldom performed in most forensic centers as part of the autopsy. A current matter of argue is who should pay the genetic test. In our opinion, public health system should assume the cost of these cardiac genetic analysis due to it is well established that genetic test in cases without conclusive cause of death help to identify the cause of death in a high number of cases. In addition, these genetic tests also help clinicians in identification of genetic carriers in family members, doing prevention of SCD in relatives at risk.

Similarly, according to recent cardiology guidelines[45], the use of molecular autopsy should be considered in the event of an unexplained sudden cardiac death with a suspicion of inherited disease. It remains unclear when to suspect an inherited cardiac disease in most cases, as death is often the first clinical manifestation in the families. According to these same guidelines, the identification of a pathogenic variant associated with long QT syndrome or with CPVT is diagnostic of the disease. Thus, taking these data into account, the use of molecular autopsy appears mandatory in order to attempt to provide a diagnosis, which may benefit the identification of family members at risk.

For that reason, the aim of our work was to evaluate whether molecular autopsy could increase the identification of a potential etiology of death. Thus, we performed genetic investigation in all cases classified as microscopic cardiomyopathy as well as in those that remained unexplained after macro and microscopic autopsy. Thirty-two samples were reclassified as cardiomyopathies after identification of positive histological alterations. Out of these 32 samples, 19 showed histological changes consistent with a diagnosis of HCM. These results agree with several studies that report that HCM is the most prevalent cardiomyopathy associated with SCD. After genetic analysis, 33.9% of cases carried at least one PPV or DM variant, most variants remaining as VUS.

In addition, we genetically analyzed 119 samples classified as negative autopsy cases. Genetic analysis identified 41.19% of cases carrying at least one PPV or DM variant, remaining most variants as VUS. This percentage is in concordance with other genetic studies performed with NGS panels in autopsy samples [46, 47]. In post-mortem studies in which only a few genes were analyzed frequencies of detection differ between 11 to 26% [21, 28, 48–51]. Our percentage is higher due to the largest number of analyzed genes. Of 119 cases classified as unexplained death, around 40% carry a potentially pathogenic variant. Concretely, in our cohort, in the population younger than 31 years of age, the percentage of potentially pathogenic rare variants was 40.4%. Some reports have established that between 10% to 25% of SUD in the adult, and up to one-third in infantile and juvenile SUD, may be explained by cardiac channelopathies[8–11]. In most of these studies, the analysis was limited to the main genes associated with channelopathies. Our higher percentage may be due to a comprehensive genetic analysis including both genes associated with channelopathies and genes associated with cardiomyopathies, recently associated with arrhythmic pathologies without any structural alteration [52, 53]. In concordance with our results, recent studies performed in post-mortem samples using NGS technology showed percentages of rare variants potentially pathogenic in 30%-40% of samples analyzed [24–26, 54–58].

Out of 119 samples, we identified 21 DM variants in 16 cases (13.44%). Of these, 5 variants were potentially responsible for LQT, and 2 for BrS. Recent guidelines recommend the use of post-mortem genetic testing in cases with clinical evidence suggesting a diagnosis of LQTS or CPVT [29, 45]. Therefore, and according to the guidelines, a diagnosis was reached as a cause of death. In addition, 51 PPV were identified in 38 cases (31.92%). While their pathogenic role cannot be fully defined, the potential for an inherited disease makes it essential to further investigate the family members for segregation. In a recent report, Bagnall et al performed a NGS analysis in a post-mortem cohort of 490 samples died suddenly between 1–35 years old[28]. They identify nearly 35% of genetic variants classified as VUS and family segregation clarify the role of nearly 15% of cases concluding that autopsy investigation combined with genetic testing and family screening is the best way to identify a conclusive cause of death in cases died suddenly.

### Overlapping diseases/genes

In our study we have identified samples classified as cardiomyopathies but carrying rare variants in genes encoding ion channels and/or associated proteins. Similarly, we have also identified samples classified as negative autopsy (potential channelopathy) but carrying rare variants in genes encoding structural proteins. Several studies have reported the potential pathophysiological mechanisms linking both entities in common genes. Thus, for example, *PKP2* (encoding plakophilin-2), is the main gene associated with AC and has been reported playing a pathogenic role in BrS [52, 53] despite additional studies in large cohorts should be performed to clarify this point [59]. In addition, alterations in *SCN5A* (encoding the sodium channel), the main gene associated with BrS, have been reported in 1–2% cases of DCM [60], and even AC [61]. In concordance with similar results from recent studies [62], this could suggest that a malignant arrhythmia could appear in early stage before a structural alteration is developed. However, further studies in larger cohorts should be performed to prove or refute this hypothesis.

### Compound/Multigenic variants

In almost 50% of samples, more than one rare genetic variant was identified, even in the same gene in some cases. Most of these rare genetic variants are at present of unknown significance. However, whether they have a role in the final risk of sudden cardiac death is unknown. Their potential effect as genetic modifiers (either detrimental or protective) of the phenotype is well accepted, but larger and more comprehensive studies will be needed to obtain conclusive data.

### Limitations

The first and main limitation already mentioned above is the lack of family members in order to perform a clinical-genetic segregation. The family segregation is crucial to clarify the role of identified genetic variants in each case/family. In addition, functional studies could help elucidate the pathogenic role of the variants in arrhythmogenesis but *in vitro* evidence of channel dysfunction associated with specific variants may not necessarily directly translate into a clinical phenotype in the complex biological environment of the human cardiovascular system. Finally, cases without any identified genetic variation could carry a defect in other genes not included in our NGS custom-panel.

## Conclusions

**Fig 6:**
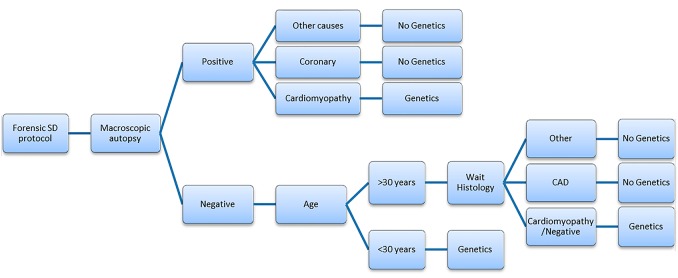
Proposal of flow chart as forensic protocol guide for Sudden Death cases. In cases less than 30 years old with a negative macroscopic autopsy or cases suspected of cardiomyopathy should be studied by genetics. Older cases must wait for histological analyses before be studied by genetics.

In a prospective cohort of 789 cases of natural death, younger than 50 years of age, we show that cardiac alterations are the most common cause of death, concretely coronary artery disease. While forensic investigation can determine the cause of death in most cases, nearly 19% of cases remain unanswered after a thorough autopsy investigation. The use of NGS genetic analysis has been advocated as an important complement to the investigation of death, and the incorporation of molecular autopsy in current guidelines attest to its value, according to the experts. The molecular autopsy may help identify the cause of death in a large percentage of cases remaining as negative after autopsy. In our cases without conclusive cause of death, we identified nearly 35% of PPV and nearly 15% of DM variants therefore reaching a conclusive diagnosis according to the guidelines. In our study we show that genetic analysis should be performed when there is a suspicion for an inherited cardiac disease after macroscopic or histological analysis, and when all tests have excluded a cause of death. Of notice, in SUD victims older than 30, it is important to exclude coronary disease by histology before proceeding with molecular autopsy, as this is the most common cause of death in that population. Our data show that before age 30, despite histological analysis should be performed in order to identify any microscopic alteration, the genetic analysis should be undertaken right away because the percentage of microscopic cardiac alterations is very low. In addition, even when histology identifies any microscopic cardiac alteration, the genetic results are a helpful complement of alterations identified in order to conclude cause of death. Consequently, we have proposed a simple forensic recommendation about molecular autopsy in Sudden Death cases (Fig 6). The evaluation of relatives should include an appropriate genetic counseling and will allow the implementation of preventive measures to the relatives at risk to prevent new cases of SCD.
