# Correction: Natural and Undetermined Sudden Death: Value of Post-Mortem Genetic Investigation

**DOI:** 10.1371/journal.pone.0171893

**Published:** 2017-02-06

**Authors:** Olallo Sanchez, Oscar Campuzano, Anna Fernández-Falgueras, Georgia Sarquella-Brugada, Sergi Cesar, Irene Mademont, Jesus Mates, Alexandra Pérez-Serra, Monica Coll, Ferran Pico, Anna Iglesias, Coloma Tirón, Catarina Allegue, Esther Carro, María Ángeles Gallego, Carles Ferrer-Costa, Anna Hospital, Narcís Bardalet, Juan Carlos Borondo, Albert Vingut, Elena Arbelo, Josep Brugada, Josep Castellà, Jordi Medallo, Ramon Brugada

There is information missing from the Funding statement. The complete funding information is as follows:

This work was supported by Obra Social "La Caixa", Fondo Investigacion Sanitaria -FIS PI14/01773- from the Instituto de Salud Carlos III (ISCIII), and Fundació Academia de Ciències Mèdiques i de la Salut de Catalunya i de Balears (ACMCB-2013). The commercial founder Gendiag S.L provided support in the form of salaries for authors CF and RB, but did not have any additional role in the study design, data collection and analysis, decision to publish, or preparation of the manuscript. The specific roles of these authors are articulated in the ‘author contributions’ section.

## References

[1] Sanchez O, Campuzano O, Fernández-Falgueras A, Sarquella-Brugada G, Cesar S, Mademont I, et al (2016) Natural and Undetermined Sudden Death: Value of Post-Mortem Genetic Investigation. PLoS ONE 11(12): e0167358 doi: 10.1371/journal.pone.0167358 27930701 10.1371/journal.pone.0167358 PMC5145162

